# Malignant Melanoma in Older Adults: Different Patient or Different Disease?

**DOI:** 10.7759/cureus.34742

**Published:** 2023-02-07

**Authors:** Daniel C Sasson, John T Smetona, Yassmin Parsaei, Marianna Papageorge, Stephan Ariyan, Kelly Olino, James Clune

**Affiliations:** 1 Division of Plastic and Reconstructive Surgery, Department of Surgery, Yale School of Medicine, New Haven, USA; 2 Division of Surgical Oncology, Department of Surgery, Yale School of Medicine, New Haven, USA

**Keywords:** recurrence, survival, mutation, age, melanoma

## Abstract

Objective

In this study, we aimed to compare the clinical outcomes between older and younger patients with melanoma and to evaluate for differences in tumor genetic makeup that might explain differences in clinical behavior between older and younger cohorts.

Materials and methods

A consecutive sample of patients diagnosed with melanoma at a single institution from 1984 to 2019 was categorized by age into younger, middle, and older cohorts. Tumor characteristics, melanoma-specific survival, and recurrence-free survival were assessed while accounting for differential follow-up and death from other causes using Kaplan-Meier analysis with log-rank testing.

Results

A total of 4378 patients were included in the study. Older patients presented with a higher incidence of T3 and T4 tumors, and a lower incidence of T1 tumors (p<0.001). The same group of patients had a lower nodal positivity at any given Breslow thickness (p<0.01). Melanoma-specific survival was lower for older patients with T2 tumors (p=0.046). There was no difference in recurrence-free survival among all age groups and tumor thicknesses (p>0.05). For patients with a given genetic profile, the melanoma-specific survival and recurrence-free survival were equivalent across ages. BRAF was the most common driver in the younger group, while NRAS and other mutations increased in prevalence as age rose.

Conclusions

Older adults have decreased melanoma-specific survival for T2 tumors and lower nodal positivity, suggesting a different pattern of metastatic progression. The mutational drivers of cutaneous melanoma change with age and may play a role in the different metastatic progression as well as the differential melanoma-specific survival across all age cohorts.

## Introduction

It is thought that melanoma portends a worse prognosis in older adults [1-4]. However, the reasons for these differences in survival and recurrence of disease, as compared to younger patients, are unclear. Determining whether the older population presents at a later stage or has innately different tumor biology can be difficult. Many studies, including the validation of the American Joint Committee on Cancer staging system in 2001 with a sample of 17,600 patients, found age to be an independent risk factor for disease-specific survival [1-5]. Nodal positivity decreases with increasing age, despite the fact that tumors tend to be thicker and wider at the time of diagnosis [2,3,5-7]. Hypotheses relating to this decrease in nodal involvement include decreased radiotracer transmission by peritumoral cells in older adults, creating in effect a false negative sampling; or, changes in the dermal lymphatic system may cause less effective trafficking of malignant cells, leading to negative nodal basins while the tumor is propagating at other distal sites, perhaps via hematogenous spread [8-10]. More recently, Kaur et al. showed that age-related changes in the extracellular matrix, particularly reduced expression of HAPLN1, reduced T cell motility and promoted tumor cell motility, thus favoring metastasis [11]. Ecker et al. have shown that changes in the extracellular matrix affected by HAPLN1 promote changes in the dermal lymphatics and that higher HAPLN1 expression correlates with increased survival [6]. They further showed that inducing HAPLN1 expression in mice increased nodal positivity and decreased visceral metastasis.

Early evidence also exists of tumors possessing different genetic profiles depending on age. BRAF has been shown to be more commonly mutated in younger patients [8]. The impact of BRAF and NRAS mutations on survival and responsiveness to therapy has been debated in the literature [8,12,13]. Differences in the genetic makeup of the tumor might explain more aggressive behavior and increased mortality in older adults.

The goal of this study is to evaluate the clinical and biological differences between younger and older melanoma patients and the effect on nodal metastases, recurrence, and melanoma-specific survival.

## Materials and methods

All procedures followed were in accordance with the ethical standards of the responsible committee on human experimentation (institutional and national) and with the Helsinki Declaration of 1975, as revised in 2008. Informed consent was obtained from all individual participants included in this study in accordance with The Code of Ethics of the World Medical Association. This review conformed to all recommendations of the Strengthening the Reporting of Observational Studies in Epidemiology Initiative and was approved by Yale University’s Institutional Human Investigation Committee (IRB/HIC) [14]. Yale University’s Joint Data Analytics Team was utilized to accurately select patients from the electronic medical records per the defined inclusion criteria. A retrospective chart review was performed of all patients who presented to a single high-volume institution with the diagnosis of malignant melanoma from 1984 to 2019. Patients presenting with acral lentiginous, desmoplastic, and mucosal melanomas were excluded due to their innately differential presentation by age.

Patients were classified into three groups according to age at the time of presentation: older (71-100 years), middle (41-70), and younger (20-40). Age greater than 70 years has previously been used as a threshold above which immune function and response to disease decrease. Thus, patients were grouped in discreet age cohorts as the efficacy of immune surveillance likely evolves over an adult's lifetime in a nonlinear manner [2,3]. When clinically indicated, tumors were sent for genetic analysis utilizing DNA microarray, including NRAS and BRAF gene assessment. Tumor characteristics including thickness, presence of ulceration, and nodal status were compared between groups. Tumor thickness was categorized by the widely-used Breslow scale, in which T1 lesions are 1 mm thick or less, T2 between 1.1 mm and 2 mm, T3 between 2.1 mm and 4 mm, and T4 more than 4 mm. Melanoma-specific survival and recurrence-free survival were assessed while accounting for differential follow-up and death from other causes using Kaplan-Meier analysis with log-rank testing. Rates of NRAS and BRAF mutations between older and younger groups were calculated. Chi-squared tests were used to assess the statistical significance of the differences in rates between groups. Bonferroni multiple-comparison correction was utilized where appropriate.

## Results

Demographics

A total of 4378 patients were included in the study: 1333 patients in the older cohort, 2572 in the middle, and 473 in the younger cohort. Median lengths of follow-up were 44, 64, and 72 months in the older, middle, and younger cohorts, respectively. Older patients presented with a higher incidence of T3 and T4 tumors, and a lower incidence of T1 tumors (p<0.001; Table 1). Older patients had a lower nodal positivity at any given Breslow thickness (p<0.01). Rates of ulceration were equivalent across age groups for T1 and T2 tumors, but older adults had higher rates of ulceration when presenting with T3 and T4 tumors.

**Table 1:** Patient characteristics per age cohort and Breslow thickness

	20–40 years, n (%)	41–70 years, n (%)	71+ years, n (%)	P-value			
				Three-way comparison	20–40 vs. 41–70	20–40 vs. 71+	41–70 vs. 71+
T1	278 (60)	1440 (59)	538 (44)	<0.001			
Nodal positive	11 (4)	21 (1)	4 (1)	0.002			
Ulcerated	2 (1)	35 (3)	15 (3)	0.214			
Recurrence in nodal basin	8 (3)	29 (2)	7 (1)	0.291			
Melanoma-specific survival				0.489	0.392	0.997	0.285
Recurrence-free survival				0.784	0.536	0.854	0.697
T2	115 (25)	518 (21)	268 (22)	0.239			
Nodal positive	21 (18)	74 (14)	14 (5)	<0.001			
Ulcerated	13 (16)	78 (18)	48 (19)	0.541			
Recurrence in nodal basin	10 (9)	47 (9)	16 (6)	0.309			
Melanoma-specific survival				0.046	0.687	0.053	0.029
Recurrence-free survival				0.342	0.175	0.125	0.807
T3	42 (9)	275 (11)	204 (17)	<0.001			
Nodal positive	18 (43)	82 (30)	37 (18)	<0.001			
Ulcerated	16 (43)	93 (37)	104 (54)	0.001			
Recurrence in nodal basin	10 (24)	42 (15)	30 (15)	0.321			
Melanoma-specific survival				0.485	0.437	0.922	0.300
Recurrence-free survival				0.060	0.897	0.224	0.027
T4	29 (6)	205 (8)	217 (18)	<0.001			
Nodal positive	20 (69)	103 (50)	51 (24)	<0.001			
Ulcerated	11 (48)	129 (69)	150 (73)	0.039			
Recurrence in nodal basin	9 (31)	51 (25)	40 (18)	0.139			
Melanoma-specific survival				0.977	0.845	0.862	0.852
Recurrence-free survival				0.940	0.846	0.678	0.855
Aggregate	464	2438	1227				
Nodal positive	67 (14)	277 (11)	103 (8)	<0.001			
Ulcerated	42 (12)	335 (16)	317 (28)	<0.001			
Recurrence in nodal basin	37 (8)	169 (7)	93 (8)	0.629			
Melanoma-specific survival				<0.001	0.748	0.002	<0.001
Recurrence-free survival				<0.001	0.441	<0.001	<0.001

Melanoma-specific survival

**Figure 1:**
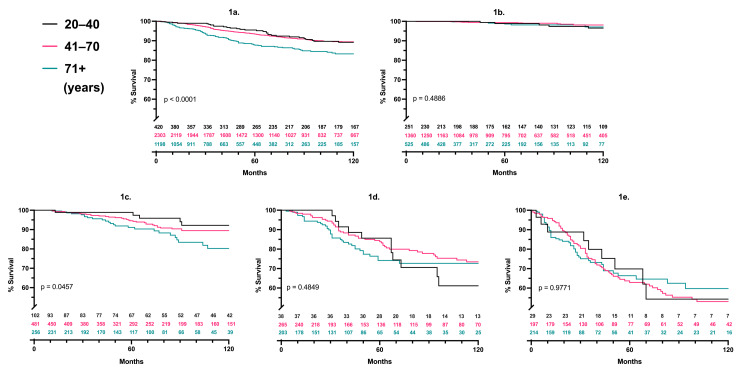
Melanoma-specific survival per age cohort and Breslow thickness P-values represent three-way log-rank comparisons; patients at risk are listed for each time point, 1a. aggregate, 1b. T1, 1c. T2, 1d. T3, 1e. T4

Kaplan-Meier analysis with log-rank testing showed melanoma-specific survival was lower for older patients with T2 tumors (p=0.046); it was otherwise statistically equivalent across age groups at a given Breslow thickness (Table 1; Figure 1b-1e). Of those who died, mortality occurred sooner in the older cohort, with a median of 2.5 years (average 3.3) until mortality versus 5.7 years (average 7.7) until mortality in the younger cohort.

Recurrence-free survival

**Figure 2:**
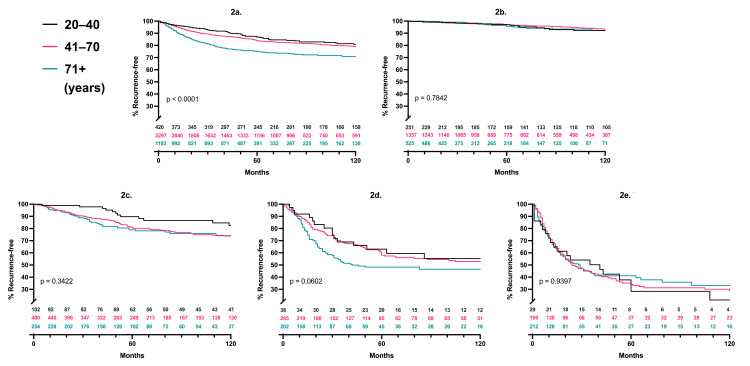
Recurrence-free survival per age cohort and Breslow thickness P-values represent three-way log-rank comparisons; patients at risk are listed for each time point, 2a. aggregate, 2b. T1, 2c. T2, 2d. T3, 2e. T4

Kaplan-Meier analysis with log-rank testing demonstrated no significant difference in recurrence-free survival for all age groups and tumor thicknesses after performing Bonferroni multiple-comparison correction (p>0.05; Table 1; Figure 2b-2e). Recurrence occurred in the nodal basin in 8% of older, 7% of middle, and 8% of younger patients. There were no statistically significant differences between age groups for recurrence in the nodal basin at any Breslow thickness. Of those who recurred, the time till recurrence was shorter in older adults, with a median time to recurrence of 1.3 years (average 1.8) for the older cohort, but 3.6 years (average 5.6) for the younger cohort.

Genetic analysis

**Figure 3:**
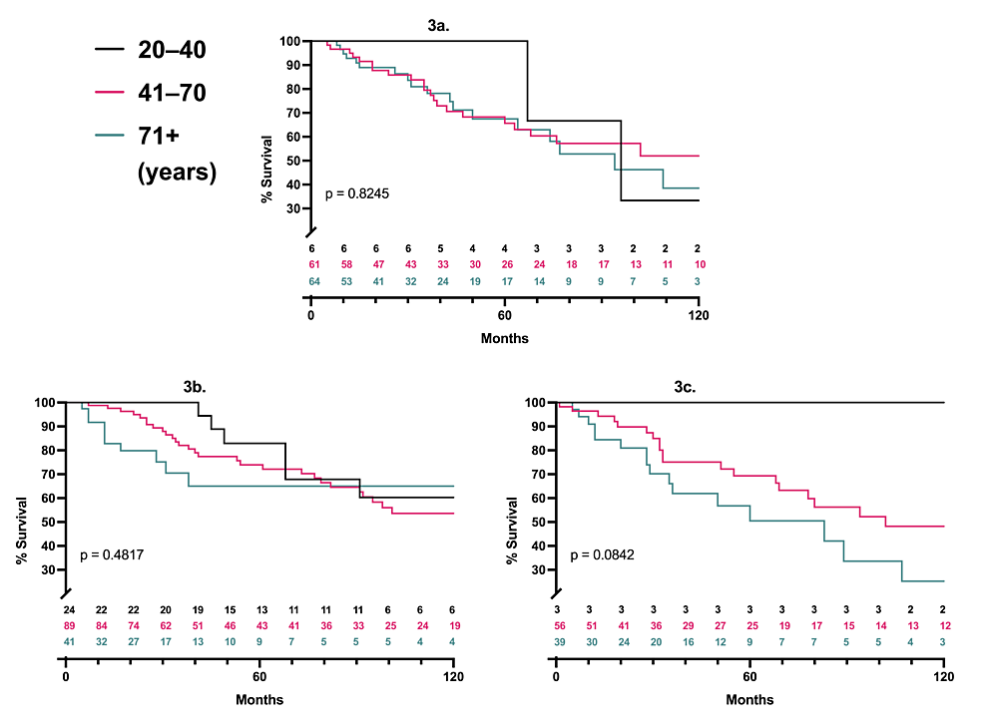
Melanoma-specific survival per age cohort and mutation status P-values represent three-way log-rank comparisons; patients at risk are listed for each time point, 3a. BRAF^wt^/NRAS^wt^, 3b. BRAF^m^/NRAS^wt^, 3c. BRAF^wt^/NRAS^m^ *wt*: wild type; *m*: mutant

**Figure 4:**
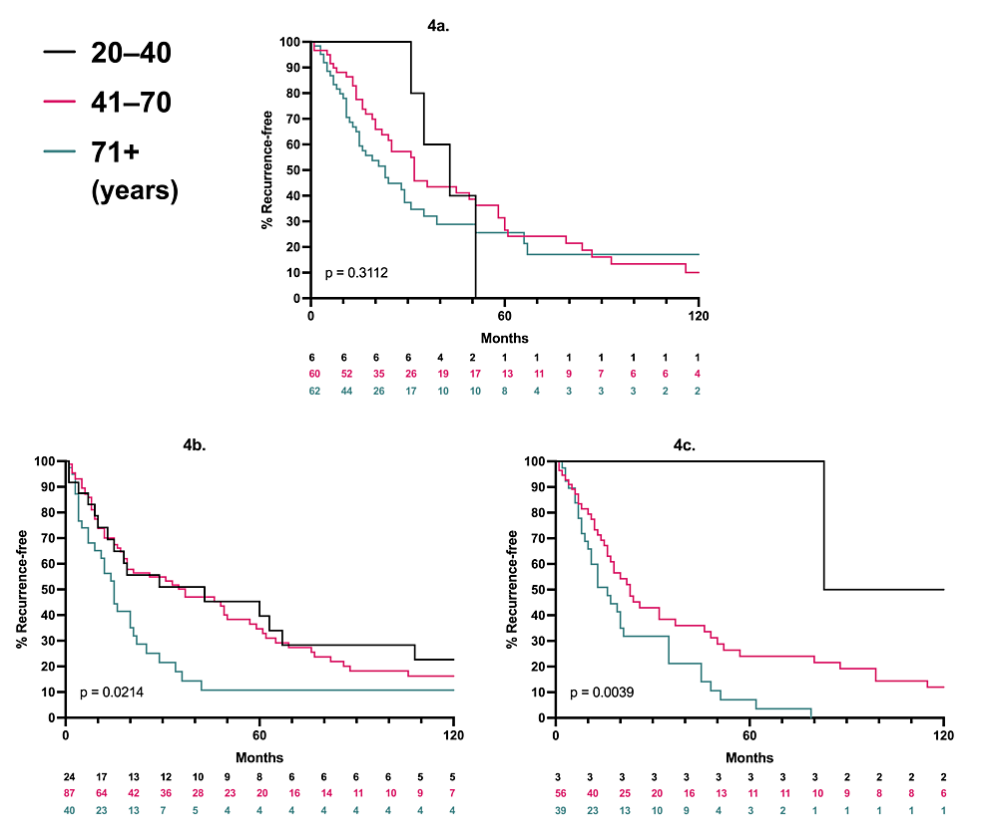
Recurrence-free survival per age cohort and mutation status P-values represent three-way log-rank comparisons; patients at risk are listed for each time point, 4a. BRAF^wt^/NRAS^wt^, 4b. BRAF^m^/NRAS^wt^, 4c. BRAF^wt^/NRAS^m^ *wt*: wild type; *m*: mutant

A total of 392 patients underwent genetic testing: 148 in the older cohort, 211 in the middle, and 33 in the younger. In the older group, 43% of patients were BRAF*^wt^*/NRAS*^wt^*. In the middle cohort, NRAS*^m^*, BRAF*^m^*, BRAF*^wt^*, and NRAS*^wt^* were all common. In the younger group, BRAF*^m^*/NRAS*^wt^* was the most common genotype (73%; Table 2). For patients with a given genetic profile, melanoma-specific survival and recurrence-free survival were equivalent across ages (Figure 3a-3c; Figure 4a-4c). For patients with T4 disease, BRAF^wt^/NRAS^wt^ tumors were less likely to have nodal positivity (Table 3). There was a non-significant association of BRAF^wt^/NRAS^wt^ tumors with lower nodal positivity for all other Breslow thicknesses as well.

**Table 2:** Patient characteristics per mutation status and age cohort *wt*: wild type; *m: *mutant

	20–40 years, n (%)	41–70 years, n (%)	71+ years, n (%)	P-value			
				Three-way comparison	20–40 vs. 41–70	20–40 vs. 71+	40–71 vs. 71+
BRAF^wt^/NRAS^wt^	6 (18)	61 (29)	64 (43)	0.003			
Nodal positive	3 (50)	18 (30)	15 (23)	0.337			
Melanoma-specific survival				0.825	0.602	0.592	0.819
Recurrence-free survival				0.311	0.792	0.279	0.195
BRAF^m^/NRAS^wt^	24 (73)	90 (43)	41 (28)	<0.001			
Nodal positive	13 (54)	51 (57)	15 (37)	0.097			
Melanoma-specific survival				0.482	0.608	0.275	0.406
Recurrence-free survival				0.021	0.354	0.048	0.014
BRAF^wt^/NRAS^m^	3 (9)	58 (27)	40 (27)	0.073			
Nodal positive	0 (0)	31 (53)	13 (33)	0.037			
Melanoma-specific survival				0.084	0.136	0.125	0.124
Recurrence-free survival				0.004	0.060	0.006	0.014

**Table 3:** Patient characteristics per Breslow thickness and mutation status *wt*: wild type; *m*: mutant

	BRAF^wt^/NRAS^wt^,n (%)	BRAF^m^/NRAS^wt^,n (%)	BRAF^wt^/NRAS^m^,n (%)	P-value
				Three-way comparison
T1	16 (35)	14 (30)	16 (35)	0.878
Nodal positive	1 (6)	4 (29)	2 (13)	0.221
T2	22 (28)	34 (43)	23 (29)	0.080
Nodal positive	3 (14)	15 (44)	7 (30)	0.056
T3	29 (28)	47 (46)	26 (25)	0.004
Nodal positive	9 (31)	27 (57)	12 (46)	0.081
T4	64 (39)	60 (37)	36 (22)	0.002
Nodal positive	23 (36)	32 (53)	23 (64)	0.018
Aggregate	131 (33)	155 (40)	101 (26)	<0.001
Nodal positive	36 (27)	78 (50)	44 (44)	<0.001

## Discussion

Tumor characteristics at presentation

Older patients present with thicker tumors and higher rates of ulceration at the time of presentation. In spite of that, there was a lower rate of nodal positivity, both when matched by Breslow thickness and in aggregate. The thicker tumors at the time of presentation raise the question of more rapid tumor growth and invasion in older adults versus an increased latency between disease onset and the initiation of medical care, or even an increased frequency of deferral of biopsy of suspicious lesions in those who may have a short life expectancy. The lower rate of nodal metastasis despite more advanced local disease is a seemingly paradoxical finding. This confirms prior data that patterns of metastasis are different in older adults, suggesting escape or dysfunction of the dermal lymphatics and a direct hematogenous spread to lung and visceral organs [1-4,6,9,15]. The fact that recurrence in the nodal basin was not more common in older adults (8% younger versus 8% older cohort in aggregate) would suggest that lower rates of nodal positivity are not due to false negative nodal biopsies at the time of tumor excision, as these would subsequently become sites of clinically evident nodal disease if they were missed. This behavior could explain the increased melanoma-specific mortality in older adults; if the lymph nodes are unable to function as immunologic surveillance centers.

Recurrence and mortality

As a whole, older adults were more likely to die from melanoma. However, when cohorts were matched by Breslow thickness, Kaplan-Meier analysis showed melanoma-specific survival was equivalent for all age groups except for T2 thickness, where it was lower in the older cohort. We do not presently have a data-driven explanation for this selectivity; a hypothesis is that less aggressive tumors may be more effectively eradicated by the younger immune system, but mortality is so low for T1 lesions that no difference is seen. T3 and T4 tumors, on the other hand, are sufficiently aggressive that the more robust younger immune system does not confer a survival benefit.

Given the differential relationship between nodal positivity and thickness in older adults, it might seem that standard TNM staging may effectively under-stage older patients since they are less likely to have nodal metastasis at a given thickness. However, their equivalent survival for T4 tumors suggests that this is not necessarily the case. It should also be noted that nearly one-third of older adults passed away due to non-melanoma causes, far in excess of that of the younger group. For this reason, the use of Kaplan-Meier analysis was critical, as otherwise, the non-melanoma mortality would decrease the apparent melanoma-specific mortality in the older cohort.

In patients with recurrence, the time till recurrence was shorter in older adults, with a median time to recurrence of only 1.3 years (average: 1.8 years) for the older group, but 3.6 years (average: 5.6 years) for the younger cohort. Similarly, melanoma-specific mortality occurred sooner in the older cohort, with a median of 2.5 years (average: 3.3 years) until death, versus 5.7 years (average: 7.7 years) until death in the younger cohort. This may suggest that older adults experience a more rapid, aggressive spread of disease when there is some component not resected at the original surgery. While a generalized weakening of immune function may contribute to this, the reduced nodal positivity may also indicate decreased antigen trapping and presentation, and consequently a reduced ability of the immune system to destroy neoplastic cells, allowing for the rapid spread of disease if it escapes the original tumor bed.

The timing of recurrence has implications for postoperative monitoring protocols. With recurrence happening so soon in older adults, and not confined to the nodal basin (only 8% recurrence rate in the nodal basin), the utility of a clinical nodal exam may be limited. For these older patients, whole-body PET scanning may be considered for those with aggressive tumor characteristics, including T2 or greater thickness or any tumor with ulceration. In the younger cohort, the fact that nearly half of the first recurrences occur more than five years from the time of diagnosis (median: four years, average 5.9 years) suggests that extended follow-up may be necessary.

Genetic data

BRAF mutations are the primary drivers of melanoma in younger patients, while in older patients, neither NRAS nor BRAF was mutated in nearly half of the patients who were tested, suggesting a polygenic or lesser-known genetic driver. This increased prevalence of BRAF mutations in younger patients has been reported previously, but it has not been shown to correlate definitively with prognosis [16].

Patients show equivalent melanoma-specific survival regardless of age when matched by tumor genotype. We also see that BRAF^wt^/NRAS^wt^ tumors exhibit lower nodal positivity for T4 tumors. Thus, we see in older adults an increased presence of a genotype that confers lower nodal positivity and higher mortality. Within the limits of our study, we cannot rule out the possibility that the mutational drivers are truly correlated with age but not causative of tumor behavior. Of note, this pattern of decreased melanoma-specific survival with BRAF^wt^/NRAS^wt^ has not been established in the literature. Studies have been conflicting to date; Fang et al. have shown data suggesting that BRAF and NRAS mutations are associated with increased failure of local control, and Jakob et al. have shown that NRAS mutation is associated with decreased survival in metastatic melanoma [8,13]. On the other hand, Pracht et al. showed a decreased response to cytotoxic and kinase-based therapy in wild-type tumors, and Carlino et al. found no impact of mutational status on survival [17,18]. Although immunosenescence has long been posited as the primary driver of decreased melanoma-specific survival in older adults, Joshi et al. have shown that older adults with advanced melanoma exhibit equivalent immune responses to the younger when treated with immunotherapy [12]. They further showed that patients with BRAF mutations were less likely to respond to immunotherapy. We do not yet have the statistical power to perform a reliable regression analysis to assess the effect of mutation status when controlling for age, tumor thickness, ulceration, and nodal status. We are now routinely sending 128 gene oncomine panels for patients with micrometastases, and expect more clarity regarding the survival impact of the different genetic drivers as our and other databases increase in power.

Limitations

This heterogeneous cohort was composed of patients with different lengths of follow-up. Deferral of nodal biopsy may have been higher in older adults due to concerns about general anesthesia and questions about the ultimate utility of this procedure in patients who may otherwise have a limited life expectancy. Only a subset of patients received genetic testing, introducing a large possible bias for the distribution of genetic mutations between groups, particularly for T1 tumors who would not routinely have nodal samples sent and therefore would not typically be candidates for immunotherapy. Maintenance of death records is highly rigorous in this database, but patients with thin T1 lesions might have been lost to follow-up at a higher rate due to less rigorous surveillance after five years of follow-up.

## Conclusions

Older adults have decreased melanoma-specific survival, possibly due to greater tumor thickness at the time of presentation. They have lower nodal positivity, suggesting a different pattern of metastatic progression. The low recurrence in the nodal basin and the rapid recurrence in older adults suggest surveillance of the nodal basin alone may not be adequate. The mutational drivers of cutaneous melanoma change with age - BRAF is the most common driver in the young, while NRAS and other mutations increase in prevalence with increasing age. BRAF^wt^/NRAS^wt^ patients have decreased melanoma-specific survival compared to those with NRAS or BRAF mutations. The mutational drivers of melanoma may play a role in the different metastatic progression as well as the differential melanoma-specific survival across age cohorts.
